# Automated Intracellular Calcium Profiles Extraction from Endothelial Cells Using Digital Fluorescence Images

**DOI:** 10.3390/ijms19113440

**Published:** 2018-11-02

**Authors:** Marcial Sanchez-Tecuatl, Ajelet Vargaz-Guadarrama, Juan Manuel Ramirez-Cortes, Pilar Gomez-Gil, Francesco Moccia, Roberto Berra-Romani

**Affiliations:** 1 Electronics Department, National Institute of Astrophysics, Optics and Electronics, 72840 Puebla, Mexico; 2 Biomedicine School, Benemérita Universidad Autónoma de Puebla, 72410 Puebla, Mexico; ajeletvargaz7@hotmail.com; 3 Computer Science Department, National Institute of Astrophysics, Optics and Electronics, 72840 Puebla, Mexico; pgomez@inaoep.mx; 4 Department of Biology and Biotechnology “Lazzaro Spallanzani”, University of Pavia, 27100 Pavia, Italy; francesco.moccia@unipv.it

**Keywords:** intracellular calcium, endothelium, cell tracking, multi-target tracking, Kalman

## Abstract

Endothelial cells perform a wide variety of fundamental functions for the cardiovascular system, their proliferation and migration being strongly regulated by their intracellular calcium concentration. Hence it is extremely important to carefully measure endothelial calcium signals under different stimuli. A proposal to automate the intracellular calcium profiles extraction from fluorescence image sequences is presented. Digital image processing techniques were combined with a multi-target tracking approach supported by Kalman estimation. The system was tested with image sequences from two different stimuli. The first one was a chemical stimulus, that is, ATP, which caused small movements in the cells trajectories, thereby suggesting that the bath application of the agonist does not generate significant artifacts. The second one was a mechanical stimulus delivered by a glass microelectrode, which caused major changes in cell trajectories. The importance of the tracking block is evidenced since more accurate profiles were extracted, mainly for cells closest to the stimulated area. Two important contributions of this work are the automatic relocation of the region of interest assigned to the cells and the possibility of data extraction from big image sets in efficient and expedite way. The system may adapt to different kind of cell images and may allow the extraction of other useful features.

## 1. Introduction

Vascular endothelium has long been considered as a homogeneous population of inert cells that forms a permeable barrier between flowing blood and surrounding tissues, regulating the exchange of nutrients, oxygen and catabolic waste at capillary level. This rather conventional view has recently given the way to a more realistic interpretation of the multifaceted role played by endothelial cells (ECs) within the cardiovascular system. The endothelium constitutes a multifunctional signal transduction surface that is now regarded as the largest endocrine organ of the body by virtue of its extension (about 350 m^2^) and ability to release a broad spectrum of paracrine mediators [1]. For instance, ECs control the vascular tone, platelet activation and aggregation and angiogenesis. Endothelial dysfunction ultimately results in severe cardiovascular disorders, such as hypertension, atherosclerosis, coronary artery disease and stroke, which may cause patient’s death [2,3].

An increase in intracellular Ca^2+^ concentration ([Ca^2+^]_i_) represents one of the most important signaling pathways whereby ECs exert their sophisticated control of cardiovascular homeostasis [4,5,6]. For instance, extracellular autacoids, such as adenosine trisphosphate (ATP) and acetylcholine, cause a robust increase in endothelial [Ca^2+^]_i_ to recruit the Ca^2+^/Calmodulin-dependent endothelial nitric oxide (NO) synthase (eNOS), thereby inducing NO release and vasodilation [7]. Likewise, endothelial Ca^2+^ signals drive the production of additional vasorelaxing mediators, such as prostacyclin I2 and hydrogen sulphide [8] and engage in Ca^2+^-dependent intermediate and small-conductance K^+^ channels to activate endothelial-dependent hyperpolarization (EDH) [9].

The intact endothelium of excised rat aorta provides an ideal preparation to investigate endothelial Ca^2+^ handling as the intracellular Ca^2+^ toolkit of native endothelium may differ from that described in cultured ECs [10,11,12]. This preparation, therefore, is suitable for both studying the Ca^2+^ response to physiological agonists, including ATP and acetylcholine [11,13,14] and investigating the Ca^2+^ response to mechanical injury that ultimately results in the process of endothelial wound healing [6,12,15]. Unraveling the signal transduction pathways engaged by physiological agonists and mechanical injury is essential to design novel therapeutic treatments aimed at restoring endothelial integrity and limiting cardiovascular diseases. Nevertheless, tracking endothelial Ca^2+^ signaling in their native microenvironment is a rather challenging task.

The ratiometric Ca^2+^-sensitive fluorophore Fura-2 is the most suitable fluorescent dye to obtain quantitative data on [Ca^2+^]_i_ and to carry out long-lasting recording due to its little photobleaching [15,16]. It has been widely used to measure intracellular Ca^2+^ signals in intact endothelium of excised rat aorta [6,11,12,13,14,15] as well as of other vascular segments [17,18].

The term calcium profile (CP) indicates a time-dependent change in the mean fluorescence emitted by a Ca^2+^ indicator, such as Fura-2, within a user-defined area, also termed region of interest (ROI), manually drawn around a single cell. The CP extraction procedure consists in collecting the mean intensity within each ROI for every frame throughout a ratio image sequence. This procedure provides a meaningful profile of the Ca^2+^ signal as a function of time when the ROI surrounds only one cell nucleus, which appears brighter as it is thicker cell area. For a conventional experiment, the CP of multiple cells is extracted from ratio image sequences acquired for hundreds or even thousands of frames (acquisition frequency 0.3 Hz): manual ROI analysis is time consuming and labor intensive and may introduce artificial signals as the position and plane of focus of the cells may change radically during an experiment. This leads the users to manually relocate the ROIs frame per frame and one by one if needed. Consequently, the process becomes tedious, impractical and even more time consuming.

The Ca^2+^ response to ATP or acetylcholine in the intact endothelium of excised rat aorta consists in a biphasic increase in [Ca^2+^]_i_, which comprises a rapid Ca^2+^ transient followed by a plateau phase of intermediate amplitude between the resting Ca^2+^ levels and the initial Ca^2+^ response [11,13,14]. Similarly, the Ca^2+^ signal induced by mechanical injury consists in a rapid Ca^2+^ peak followed by a long-lasting decline towards the baseline in the majority ECs adjacent to the lesion site. The remaining fraction of cells in the first row also displayed periodic Ca^2+^ oscillations which overlapped the decay phase and were initiated after the initial Ca^2+^ spike. Furthermore, repetitive Ca^2+^ spikes and heterogeneous Ca^2+^ signals were detected also in ECs further back from the wound edge (see Supplementary Material in Reference [15]). As discussed elsewhere [6], injury-induced intra- and intercellular oscillations in [Ca^2+^]_i_ might play a key role in healing damaged endothelium by inducing surviving ECs to proliferate and migrate in order to cover the denuded membrane basement.

Despite the obvious importance of recording the Ca^2+^ signal in all the cells that lie beyond the injury edge, several technical difficulties prevented us from successfully accomplishing this task. The main technical difficulties were: (1) cell selection was performed by manual ROI drawing around the cells: in the typical microscope visual field of intact endothelium at 40× magnification, around 100–150 cells can be visualized, this resulted in a time consuming cell selection; (2) as endothelial borders on in situ endothelium were not clearly identifiable, a ROI might include part of the cytoplasm of adjacent cells, so a special attention should be taken in order to draw ROI very close to the nucleus area, increasing the ROI time selection and error possibilities; (3) as ECs of intact vessels are located over a thick layer of vascular smooth muscle that contract or relax in response to vasoactive substances released by the chemical or mechanical stimulated endothelium, this results in the movement of ECs during the experiment protocol in such a way that ROI must be repositioned manually throughout the experiment and according to the specific trajectory of each endothelial cell; (4) under the mechanical injury procedure, cells that are damaged from the glass micro-pipette tip (see Section 4. Materials and Methods), immediately loose Fura-2 fluorescence and disappear from the microscope field (here defined as OFF-LINE cells, in contrast to ON-LINE which are defined as the cells present); in addition, as shown in Reference [15], they are able to incorporate ethidium bromide, a fluorescent molecule unable to cross an intact plasma membrane and therefore indicative of damaged/dead cells. A wrong interpretation of data will occur if the CPs from ROIs that enclose those damaged/dead cells are mistakenly taken in to account. It should also point out that the simple response to Ca^2+^-releasing agonists, such as ATP and acetylcholine, is hampered by the fact that bath application of these agonists stimulates the underlying layer of vascular smooth muscle cells (VMSCs). The following contraction of the muscular component of the artery wall causes many ECs to transiently disappear during some frames because of movement artefacts, thereby emitting less fluorescence and introducing a false negative in the Ca^2+^ signal.

In this work, a proposal to automate the CP extraction process is presented. Taking advantage from the slow cell movement, a tracking scheme based on intersections was implemented, which proved to be a simple and fast solution to avoid the movement artefact of the endothelial monolayer in situ caused by either physiological (due to VSMC contraction) of mechanical (due to the direct damage of the micro-pipette tip) stimulation. Although overlap-based tracking is a straightforward solution in many cases [19,20], problems related to partially missing data require the use of complementary approaches for robustness improvement. In this work, a Kalman filter is incorporated into the proposed system as a computational tool to keep track of cells in movement even when they momentarily vanish. Kalman estimation has been successfully used for object tracking in several applications [21,22,23]. The position, velocity and acceleration of the cells are estimated in every iteration to be able to predict the position of the current cell in the case it has disappeared.

## 2. Results

The intracellular CPs are extracted with the aim of modeling the endothelium behavior under different stimuli. With that purpose in mind, the application was tested with several image sequences obtained from two principal types of experiments: Endothelium under chemical (ATP) stimulus and Endothelium under mechanical stimulus. First, we will illustrate the procedure that we exploited to automate CP extraction in in situ ECs and then get into the details of this novel algorithm.

**Figure 1:**
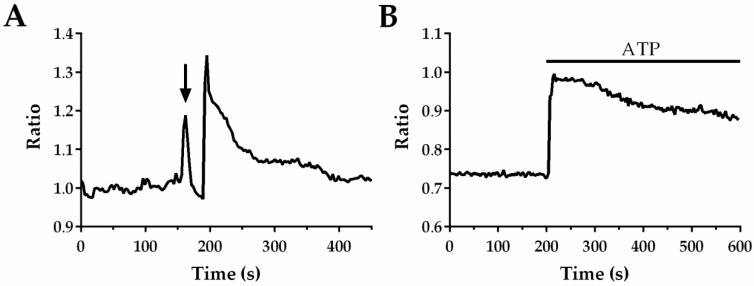
Ca^2+^ profiles from in situ endothelial cells obtained under two different stimuli (**A**) Damaging rat aorta inner wall by using the tip of a glass micro-pipette evokes a long-lasting Ca^2+^ signal in endothelial cells (ECs) located at the injury site. The arrow indicates the time when injury was performed; (**B**) Intracellular Ca^2+^ transient evoked by ATP 300 (µM) in in situ ECs from rat aorta. Calcium profile (CPs) were obtained from one single cell using the automated CP extraction method.

Online Supplementary Video 1 shows the typical experimental procedure to automate CP extraction in in situ ECs that undergo a mechanical injury imposed by the tip of a glass micro-pipette, as described in Materials and Methods. The upper left movie shows the inner wall of the aortic rings loaded with the Ca^2+^-sensitive fluorophore, Fura-2, which was fully covered by fluorescent ECs. At frame 0, an example of the adaptive segmentation outcome is shown, bordering all ECs visualized in the microscope field. For illustrative proposes, a single EC was selected and a orange ROI was automatically drawn around it in order to extract its CP and tracking its frame to frame position during the experiment (see Video 1 at frame 3). The CP was simultaneously measured and illustrated in the bottom part of the movie. Scraping the lumen of the aortic ring with the tip of a glass micro-pipette (approximately at frame 63) caused a rapid Ca^2+^ peak following by a long-lasting decline to the baseline in the ECs adjacent to the injury site, such as the EC enclosed by the orange ROI. As it can be observed on the “Position graph (upper right movie)” when the injury occurs, a drastic position change takes places for the cell surrounded by the tracking ROI. However, despite the clear movement of the EC, the automated data processing procedure enables the ROI to remain positioned over the cell of interest, which leads to a careful measurement of the Ca^2+^ signal. In Figure 1A, a typical CP obtained by the automated tracking system is shown, where the arrow indicates when mechanical injury was performed.

Online Supplementary Video 2 shows a similar procedure used to track an EC in its native microenvironment stimulated by the application of 300 µM of ATP at frame 63. Under this experimental condition, a smallest cell movement was observed. The CP obtained by using the automated CP extraction algorithm is showed in Figure 1B. In the following paragraphs, we will discuss how our Automatic Tracking procedure enables to record the true Ca^2+^ signals induced by the two distinct stimuli without missing any relevant information.

The blocks that will be presented in the Materials and Methods section were merged to conform a functional, easy and user-friendly software application. It is worth pointing out that the proposed blocks are the fundamental components of this proposal to automate the methodology for [Ca^2+^]_i_ profiles extraction. To achieve the functionality of the software application, these components were connected with control flows as shown in the high-level flowchart, provided in Flowchart file as Supplementary Material. Programming provides certain degree of freedom, therefore, the implementation of the blocks and their integration into a system may be enhanced as required. The application was built using the Matlab^®^ GUIDE.

### 2.1. ATP Stimulus

**Figure 2:**
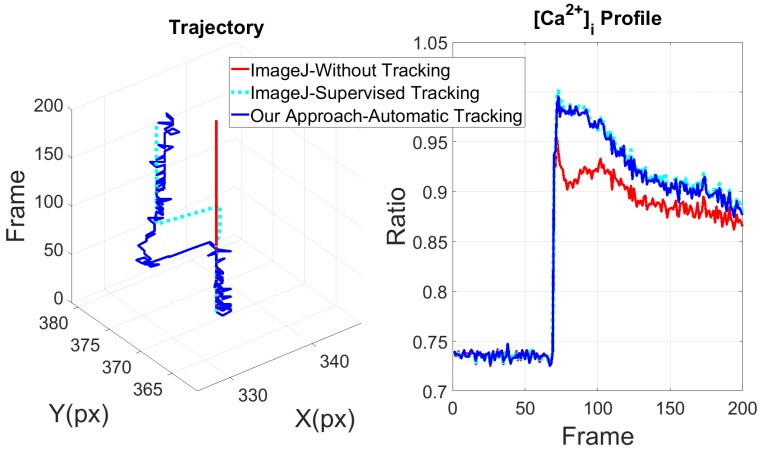
(**Left**), shows the cell trajectory, cell position in X and Y axes respectively, measured in pixels (px) and compared between ImageJ without tracking (red line), ImageJ with manually tracking (cyan line) and trajectory obtained with our automated tracking proposed system (blue line); (**Right**), shows an example of CPs calculated in each condition previously described.

This is a chemical stimulus that has been delivered as described in Materials and Methods. The dimensions of the images are 640 × 480 pixels (px) and they have *. TIFF format. Application of 300 µM ATP to in situ endothelium from rat aorta is able to evoke a biphasic increase in [Ca^2+^]_i_, as shown in Figure 2 right (blue line, data obtained with Automatic Tracking approach). However, ECs undergo a little displacement and intensity variations when exposed to bath application of the agonist. For comparison purposes, fifteen cells were selected randomly and their intracellular CPs were obtained using the proposed system and ImageJ program, manually repositioning the ROI every time the cell moves outside of it (ImageJ-Supervised Tracking). The mean square error (MSE) between both obtained data sets is shown in Table 1. Please note that MSE is insignificant compared with the mean of its respective CP extracted with our automated approach ($\bar{\mathrm{CP}}$). Although small movements were registered in the cells trajectory, the kinetics of CPs are very similar and the MSE is small, as was expected. This observation demonstrates that this approach, which consists in the simple bath application of the agonist, does not generate significant artifacts and does not induce significant VSMC contraction. It should, however, be pointed out that the magnitude of the initial peak, which is due to Ca^2+^ release from the endoplasmic release, is significantly larger than the amplitude measured without tracking (red line). Cyan line, represent the CP obtained by using ImageJ-Supervised Tracking. In this particular experiment, the single ROI was repositioned at least 3 times along the experiment. As expected, the CP obtained by ImageJ-Supervised Tracking method, overlaps with the CP obtained with our automated approach. This observation reinforces the notion that automatic tracking is recommendable to speed off-line analysis and to gather all the information content of the Ca^2+^ signal.

In order to carry on performance evaluation of the system, the density of tracked cells per frame ρ was proposed as figure of merit. The indicator represents an instantaneous measurement of the performance at a given frame i and it is defined in (1).

(1) $$\rho_{i}=100\times \frac{\mathrm{Number}\;\mathrm{of}\;\mathrm{ONLINE}\;\mathrm{cells}\;\mathrm{at}\;\mathrm{frame}\;i}{\mathrm{Number}\;\mathrm{of}\;\mathrm{binary}\;\mathrm{objects}\;\mathrm{at}\;\mathrm{frame}\;i}$$

The fluorescence of ECs may decrease or be lost, temporarily or definitively, along a given experiment. A parameter defined as reliability limit (RL) was introduced to provide to the user the possibility to control how many times a cell is allowed to disappear consecutively from the microscope field before it is considered as unreliable to be measured and marked with OFF-LINE status. Health science experts are able to set RL based on their expertise. Usage model of RL is detailed in Section 4.4.

Image sequences of two different ATP experiments with 200 images per excitation wavelength were analyzed. All the detected cells were selected to test the performance of this approach with several reliability limits (RL). The average results are shown in Table 2 and the average number of detected and selected cells was 190.

It can be seen that ρmax exceeds the 100%, which is due to the fact that a few cells disappear in some frames thereby causing a mismatch between a given number of cells and a higher number of ROIs than cells. On the other hand, the big amount of noise in some frames produces more binary blobs than ROIs.

### 2.2. Mechanical Stimulus

In this experiment, a region of the tissue is damaged with the tip of a glass micro-pipette. Detailed experimental procedure is described in Section 4. Materials and Methods. The image sequences are in *.TIFF format with a dimension of 768 × 576 pixels.

**Figure 3:**
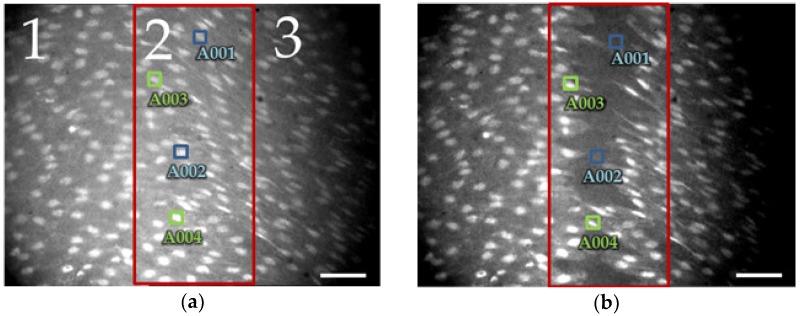
Images from mechanical stimulus experiment. (**a**) Before and (**b**) after the tissue injury. Suggested zones for the analysis (1, 2 and 3). Blue ROIs, A001 and A002 in (**a**) shown cells in the injured zone that were damaged by the tip of glass micro-pipette and lost Fura-2 fluorescence and then die and disappeared ((**b**) blue ROIs, A001 and A002); these ROIs were marked as OFF-LINE cells. Green ROIs, A003 and A004, enclosed cells adjacent to the injured area, which have partially lost the contact with neighboring ECs, but preserved Fura-2 fluorescence after injury ((**b**) green ROIs, A003 and A004); considered as ON-LINE cells. Bar length is 40 µm.

Three zones were considered in the image as shown in the Figure 3a and three cells were selected per zone. Zone 2 represents the area onto which the mechanical lesion is imposed, whereas zones 1 and 3 represent the most proximal regions to the damaged site. The fluorescence and position of cells in zones 1 and 3 display a more stable behavior than cells in the zone 2. Some cells of the inner zone (red rectangle on Figure 3a,b) die and then disappear, please see the transition from Figure 3a to Figure 3b; this is reflected as an OFF-LINE status (i.e., blue A001 and A002 ROIs).

**Figure 4:**
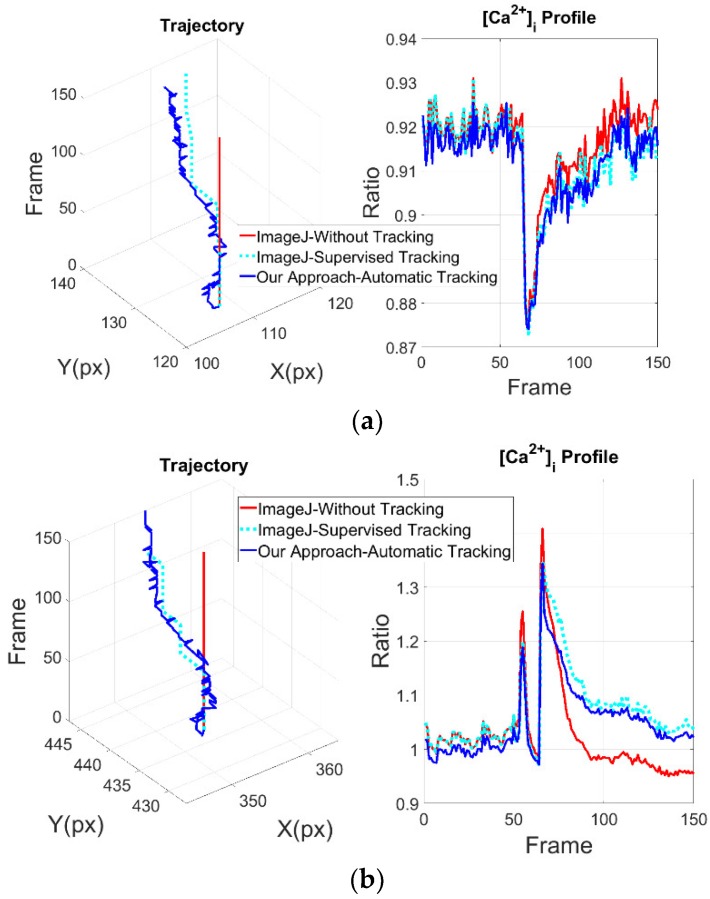
Results per defined zones due to mechanical stimuli: (**a**) Results obtained from zones 1 and 3; (**b**) Results obtained from zone 2. (**Left**), shows trajectories of cells, i.e., calculated position on X and Y axes respectively, measured in pixels (px). (**Right**), shows typical recordings of CP analyzed by: ImageJ without tracking (red line); with manually tracking (cyan line) and automated tracking proposed system (blue line).

As the cells moves slightly in the outer zones (1 and 3), it can be seen that the intracellular CPs obtained with ImageJ and with the proposed approach are very similar (Figure 4a). The results obtained from zone 2 show a more notable difference in the structure of the CPs and in the trajectory of the cells (Figure 4b).

Based on the mean squared difference of the results obtained from zones 1 and 3, indicated in Table 3, the proposed scheme delivers consistent results as shown in the previous subsection. While the results of the zone 2 highlights the importance of tracking block since the structure of CPs changes dramatically, it is noteworthy that there are patterns of intracellular CPs associated to different physiological functions. It turns out that a better profile extraction may help a better understanding of cellular behavior [15]. Accordingly, the CP of zone 2 provided by the proposed approach shows a clearly discernible plateau following the initial peak that is missed by ImageJ without tracking. This signal, therefore, reflects the true dynamics of the Ca^2+^ response induced by mechanical stimulation in ECs directly exposed to mechanical injury. The difference in the waveform of the Ca^2+^ signal is rather important, as the Ca^2+^ plateau has long been known to recruit a number of Ca^2+^-sensitive decoders involved in cell proliferation and migration, which are the crucial steps for the regeneration process.

As for ATP experiments, two mechanical stimulus experiments with 200 frames per wavelength were used to test the approach. The average results are in Table 4 where the average number of selected cells was 283. As expected, compared to the results of ATP experiments, a lower performance can be which is caused by the more random and more noticeable movement of the cells.

## 3. Discussion

A multi-target tracking system to automate the intracellular CP extraction from fluorescence image sequences was designed and implemented. The approach was based on logical intersections supported by Kalman estimation which was fed with a constant acceleration model.

A figure of merit was proposed to measure the performance and the system was tested using several image sequences from two different types of experiments, one of them (i.e., ATP experiment) showed the extracted data consistency and the other one (i.e., mechanical lesion) showed the actual need of the cell tracking.

The results allow us to conclude that an important contribution of this work is the automatic relocation of the regions of interest based on statistical measurements which favors massive data extraction and time saving during the analysis. Some appropriate technological improvements of the acquisition systems, for example, faster sample rate, combined with this approach may ensure a meaningful performance improvement, that is, more frames equals more intersections. Finally, the simplicity and efficiency of the proposed approach would allow a real-time application.

In conclusion, this novel algorithm provides an additional tool to accelerate the analysis of the hundreds of endothelial Ca^2+^ signals arising within the intact wall of excised vessels in response to physiological stimuli and to uncover the real dynamics of Ca^2+^ waves at the edge of the lesion site. This approach will be exploited to get more insights into injury-induced intra- and intercellular Ca^2+^ waves, that could in turn lead to the identification of alternative targets to prevent endothelial dysfunction induced by the implantation of medical devices, such as stents and angioplasty, or turbulent blood flow.

## 4. Materials and Methods

### 4.1. Dissection of the Aorta

Male Wistar rats aged 2–3 months were provided by the Benemérita Universidad Autónoma de Puebla animal core facilities, where they were kept under conditions of constant environmental temperature and exposed to dark light cycles of 12 h, with water and food *ad libitum*. All the experimental procedures on animals were performed according to protocols approved by the University Animal Care and Use Committee, identification code: BERRSAL71, 18-05-2017. The procedure for obtaining in situ ECs from rat aorta, was developed by Berra-Romani and Cols., 2004 [24] and it is briefly described below. The day of the experiment rats were sacrificed with an overdose of intraperitoneal ketamine-xylazine solution, 0.2 mL per 100 g of weight; subsequently they underwent to an anterior thoracotomy to expose the aortic arch and the heart. The heart was extracted, to cannulate and perfuse the aorta with a physiological salt solution (PSS). Thoracic and abdominal aorta were dissected out and placed in a Petri dish with PSS at room temperature. Using a stereomicroscope (Nikon SMZ-2T), the connective and fatty tissues surrounding the aorta were removed. Subsequently the aorta was cut into ~5 mm wide rings, stored in PSS at room temperature (22–24 °C) and used within 5 h. Using a microdissection scissors, the aortic rings were carefully cut to open and care was taken to avoid any damage to the endothelium and obtain aortic strips with intact endothelium.

### 4.2. [Ca^2+^]_i_ Measurements

The aortic strips with intact endothelium were bathed in PSS with 16 µmol Fura-2/AM for 60 min at room temperature, washed for 30 min to allow intracellular de-esterification of Fura-2/AM and fixed (with the luminal face up) to the bottom of a Petri dish covered by inert silicone (Silgard^®^ 184 Silicone Elastomer, Down Corning, MI, USA) by using four 0.4 mm diameter stainless steel pins. In situ ECs were visualized by an upright epifluorescence Axiolab microscope (Carl Zeiss, Oberkochen, Germany), equipped with a Zeiss 40× Achroplan objective (water-immersion, 2.05 mm working distance, 1.0 numerical aperture). To monitor the [Ca^2+^]_i_, ECs were excited alternately at 340 and 380 nm and the emitted light was detected at 510 nm. A neutral density filter (optical density = 1.0) was coupled to the 380 nm filter to approach the intensity of the 340 nm light. A round diaphragm was used to increase the contrast. A filter wheel (Lambda 10, Sutter Instrument, Novato, CA, USA) commanded by a computer positioned alternately along the optical path the two filters that allowed the passage of light respectively at 340 and 380 nm. Custom software, working in the LINUX environment, was used to drive the camera (Extended-ISIS Camera, Photonic Science, Millham, UK) and the filter wheel and to measure and plot in real time (on-line) the fluorescence from ~80–100 drawn manually rectangular “regions of interest” (ROI) enclosing one single cell. [Ca^2+^]_i_ was monitored by measuring, for each ROI, the ratio of the mean fluorescence emitted at 510 nm when exciting alternatively at 340 and 380 nm (shortly termed “ratio”). An increase in [Ca^2+^]_i_ causes an increase in the ratio. Ratio measurements were performed and plotted on-line every 3 s. The on-line procedure allowed monitoring of [Ca^2+^]_i_ at the same time as micro-pipette injury. The fluorescence images obtained at 340 nm and 380 nm were stored on the hard disk and data analysis was carried out later as described in data analysis section. The experiments were performed at room temperature (20–22 °C) to limit time-dependent decreases in the intensity of the fluorescence signal.

### 4.3. ATP Stimulation of in Situ Endothelial Cells

Medium exchange and administration of ATP (300 µM) was carried out by first removing the bathing medium (2 mL) by a suction pump and then adding the desired solution. The medium could be substituted quickly without producing artifacts in the fluorescence signal because a small meniscus of liquid remained between the tip of the objective and the facing surface of the aorta.

### 4.4. Mechanical Disruption of in Situ Endothelial Cells

As shown in Reference [15], aortic endothelium was injured under microscopic control by means of a glass micro-pipette with a tip of about 30 μm diameter, driven by an XYZ hydraulic micromanipulator (Narishige Scientific Instrument Lab., Tokyo, Japan). The electrodes were fabricated using a flaming-brown micropipette puller P-1000 (Sutter Instruments, Novato, CA, USA). Images of Fura-2-loaded ECs, together with numbered ROI, were taken before the lesion, to identify the cells facing the injury site. The microelectrode was first positioned almost parallel and very near to the endothelium surface. Then it was moved downward, along the z-axis, until the electrode tip gently touched the endothelium and moved horizontally across the visual field to scrape 1–3 consecutive rows of ECs.

#### 4.4.1. Chemicals

Fura-2/AM was obtained from Molecular Probes (Molecular Probes Europe BV, Leiden, The Netherlands). All other chemicals were purchased from Sigma.

#### 4.4.2. Solution

The composition of the PSS expressed in mmol/L was: NaCl (140), KCl (4.7), MgCl2 (1.2), Glucose (6), CaCl2 (2.5) and HEPES (5). Solution was titrated to pH 7.4 with NaOH.

#### 4.4.3. Data Analysis

**Figure 5:**
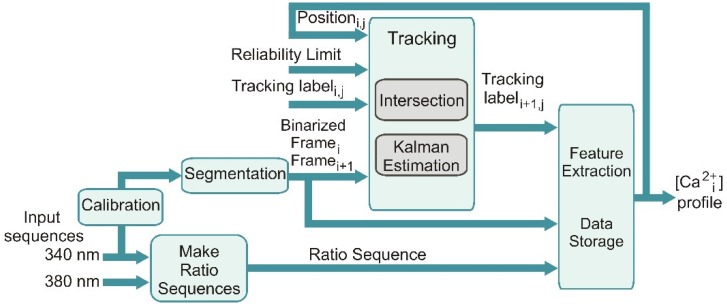
Block diagram of the proposed solution.

Endothelial Ca^2+^ imaging data were processed using an automated data processing procedure. The data processing procedure approach is represented in the block diagram shown in Figure 5. As the first step, the fluorescence images obtained at 340 and 380 nm are separately loaded; thereafter, a calibration stage is inserted to determine the parameters used by the image segmentation.

The sequence of ratio images is concurrently generated (Make Ratio Sequences). A noise reduction and an adaptive thresholding processes are performed over an image sequence. The resulting binary images are taken as the basis for the tracking scheme based on logical intersections; the tracking scheme is, in turn, enhanced with state estimation by using a Kalman filter. Feature extraction of the ROI and the storage of useful data are performed in the last sub-block.

### 4.5. Ratio Sequence Block

Fura-2 indicator is excited with λ = 340 nm and λ = 380 nm wavelengths and its fluorescent emissions are both monitored at λ = 510 nm; therefore, two image sequences are generated as experimental data, that is, an image sequence per excitation wavelength.

In this module the pixel to pixel ratio, λ = 340 nm over λ = 380 nm excitation wavelength images, is carried out for each frame to generate the Ratio image sequence. At this point, there are three image sequences available: two 8-bit resolution (uint8) *.TIFF and one float type image sequences. The used arrays have *m* × *n* × *k* dimensions, where *m* = 576 and *n* = 768 are the images height and width, respectively and *k* is the number of images per sequence. It is noteworthy to remember that CP are intensity measurements extracted from the ratio images.

As stated above, Fura-2 has a maximum fluorescence when it is excited with 340 nm wavelength, so the contrast of these images is usually better than the contrast of images obtained with 380 nm excitation. Therefore, the 340 nm image sequence was taken as the basis for the calibration, segmentation, tracking and feature extraction blocks. To guarantee the integrity of Ca^2+^ measurements, the 340 nm image sequence is modified only right after the ratio sequence was generated. The 380 nm image sequence was used only to generate the ratiometric image sequence.

### 4.6. Calibration Block

In this stage, the user manually selects several sample cells, ROIs, from a λ = 340 nm frame. Then the average of the selected ROIs areas, defined as µ*_Areas_*, is used to calculate the window size *w* to be applied on the next stages, that is, filtering and binarization.

As this is based on area statistics, the more ROIs are selected the better *w* approximation can be computed. This stage allows the system to be adapted to different cell sizes. Based on the assumption that selected cells are bounded by *w* × *w* squares, the proposed window size is defined as *w* = [*a* × √µ*_Areas_*], where *a* is scalar.

**Figure 6:**
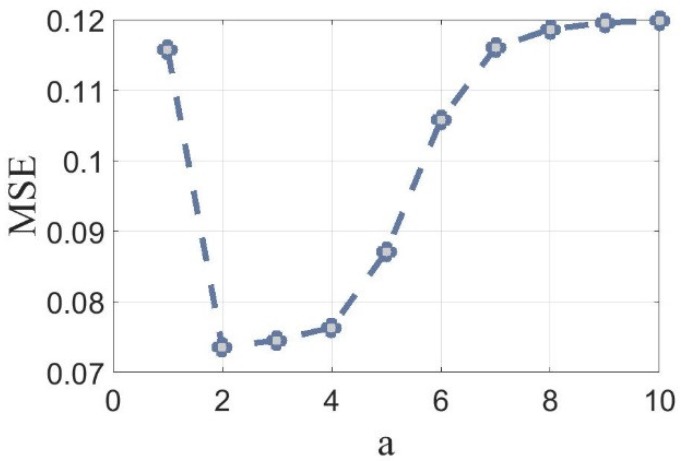
Mean square error (MSE) of binarization and ground truth segmentation for different *a* value.

A set of images was manually segmented by health science experts, aka ground truth segmentation. The same images were also segmented and conditioned automatically with algorithm presented in Section 4.7 and different *a* values. Then the mean square error between corresponding couples of images was calculated. To simplify the calculation, the binarized images were converted to double data type to use the mmse Matlab built in function. As shown in Figure 6, the minimum mean square error was obtained when *a* = 2.

### 4.7. Segmentation

In order to reduce noise, preserve the sharp high frequency detail and enhance the contrast of the images, a median filter in cascade with linear histogram stretching was used before the thresholding process. The median filter square window size was set to *w_median_* = *w*/8.

Adaptive binarization based on local statistics was taken as a solution to work with non-uniform illumination images. Since the efficiency of integral image representation has been proven for mean and variance calculation [21], it was used to implement the Niblack technique [22], whose threshold is defined in (2).

(2) $$T\left(x,y\right)=m\left(x,y\right)+k_{T}s\left(x,y\right)$$

where *m* and *s* stand for local mean and local standard deviation, respectively; the scalar gain was experimentally set to *k_T_* = 1.

The last conditioning sub stage was composed of binary image opening with disk of radius 3 as structural element; then, the holes in binary blobs were filled up and finally a dilation with radius 1 disk was applied.

### 4.8. Tracking Block

The purpose of this block is to follow the selected cells through the image sequences, this task is performed using labels associated to the cells, termed tracking labels. A cell tracking scheme based on intersections was implemented, which proved to be a simple and fast solution, with the drawback of missing cells in subsequent images due to several conditions. In consequence, cell tracking was enhanced using Kalman estimation. The goal of health science experts is to understand the behavior of vascular endothelium, through its Ca^2+^ signals and to discover its regeneration mechanisms under some conditions and stimuli. When endothelium is stimulated, many cells transiently disappear during some frames because of movement artefacts, thereby emitting less fluorescence. In this situation, it is very probable to detect no intersection. Kalman filtering has been successfully used for object tracking in several applications [21,22,23]. In this work, a Kalman filter is incorporated into the proposed system as a computational tool to keep track of cells in movement even when they momentarily vanish. The position, velocity and acceleration of the cells are estimated in every iteration to be able to predict the position of the current cell in the case it has disappeared.

A counter, per cell, called *reliability counter* is compared with RL to determine the cell status, that is, ONLINE or OFF-LINE. When a cell is selected by the user, it is surrounded by a ROI and the software application sets ONLINE status as an initial condition. Only ONLINE cells are tracked to avoid loss of performance. As of this point, when the *position* of an object is mentioned, it is referring to its *centroid*.

#### 4.8.1. Logical Intersections Approach

**Figure 7:**
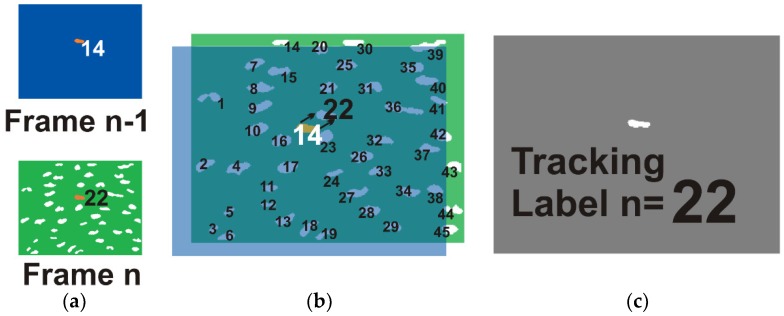
The intersection process to track cells, from frame n-1 to frame n, is illustrated. (**a**) The kernel of a selected cell with track_label = 14 in frame *n* − 1 (blue background and orange highlighted) and the binarized and labeled frame *n* are the input data, notice that the corresponding label for selected cell will be 22; (**b**) Kernel and binary image are overlapped to show the intersection process, which is a logical and operation between them; (**c**) The outcome is a new kernel, in this case only one object was generated by the intersection; based on its position in frame *n* the new track_label value is updated to 22. This iterative process is performed per each selected cell (ROI) and each frame.

As tracking block is based on logical intersections, that is and operations, it works iteratively with two frames of binarized and labeled images, *f_n_*_−1_ and *f_n_*. The main goal is to identify the selected cells from *f_n_*_−1_ to *f_n_* through the correspondence of the labels, which is termed track_label. A kernel is obtained for each selected cell with the intersection of the current cell with the *f_n_*. When intersection detects only one object, the centroid of that object is obtained in order to get the tracking label in *f_n_*. When more than one or no object is detected, the prediction equations of Kalman filter are used to estimate the position and to find the new label for the current cell in *f_n_*. This process is illustrated in Figure 7.

Once the new tracking label is found, a block termed *store data* is responsible of storing the position and intensity measurement for each cell and resets the reliability counter. If no label is detected with intersections neither with Kalman estimation, it is assumed that the cell disappeared in that frame and the reliability counter value is increased by one. When this counter is equal to the user defined RL, the tracking is considered as unreliable and the status of the current cell is changed to OFFLINE and its new tracking label is no longer searched. The block is initialized with *f_n_*_−1_ = 1 and *f_n_* = 2 and finishes with *f_n_*_−1_ = *n* − 1 to *f_n_* = *k*.

#### 4.8.2. Kalman Estimator

Although the cell motion is relatively slow, its randomness entails that the tracking based on intersection may detect in the kernel more than one or none object due to the uncertainty in the target trajectory or acceleration at any given time. Since it is extremely complex to find an exact mathematical model to describe the motion of cells, for tracking purposes, it was assumed that cells have a constant acceleration motion. The deterministic description for two dimensions in state space model is shown in (3).

(3) $${\left[\begin{array}{c} \begin{array}{c} x\\ \dot{x} \end{array}\\ \begin{array}{c} \ddot{x}\\ y \end{array}\\ \begin{array}{c} \dot{y}\\ \ddot{y} \end{array} \end{array}\right]}_{n+1}=\left[\begin{array}{cccccc} 1 & T & \frac{T^{2}}{2} & 0 & 0 & 0\\ 0 & 1 & T & 0 & 0 & 0\\ 0 & 0 & 1 & 0 & 0 & 0\\ 0 & 0 & 0 & 1 & T & \frac{T^{2}}{2}\\ 0 & 0 & 0 & 0 & 1 & T\\ 0 & 0 & 0 & 0 & 0 & 1 \end{array}\right]{\left[\begin{array}{c} \begin{array}{c} x\\ \dot{x} \end{array}\\ \begin{array}{c} \ddot{x}\\ y \end{array}\\ \begin{array}{c} \dot{y}\\ \ddot{y} \end{array} \end{array}\right]}_{n}+{\left[\begin{array}{c} \begin{array}{c} \frac{T^{3}J_{x}}{3}\\ \frac{T^{2}J_{x}}{2} \end{array}\\ \begin{array}{c} TJ_{x}\\ \frac{T^{3}J_{y}}{3} \end{array}\\ \begin{array}{c} \frac{T^{2}J_{y}}{2}\\ TJ_{y} \end{array} \end{array}\right]}_{n}\;z=\left[\begin{array}{c} \begin{array}{ccc} \begin{array}{cc} 1 & 0 \end{array} & \begin{array}{cc} 0 & 0 \end{array} & \begin{array}{cc} 0 & 0 \end{array} \end{array}\\ \begin{array}{ccc} \begin{array}{cc} 0 & 0 \end{array} & \begin{array}{cc} 0 & 1 \end{array} & \begin{array}{cc} 0 & 0 \end{array} \end{array} \end{array}\right]{\left[\begin{array}{c} \begin{array}{c} x\\ \dot{x} \end{array}\\ \begin{array}{c} \ddot{x}\\ y \end{array}\\ \begin{array}{c} \dot{y}\\ \ddot{y} \end{array} \end{array}\right]}_{n}$$

where *J_n_* is a random change in acceleration, a.k.a. jerk, occurring between time *n* and *n* + 1. The random jerk *J_n_* has the auto correlation function given by (4) and it is characterized as white noise.

(4) $$J_{n}{\bar{J}}_{m}=\{\begin{array}{ll} \sigma_{J}^{2} & \mathrm{for}\;n=m\\ 0 & \mathrm{for}\;n\neq m \end{array}$$

With the sampling period of the experimental data, *T* = 3, the observability matrix has full rank, that is, rank = 6, therefore the proposed system model is observable and the Kalman filter can work correctly. Assuming statistical independence between the coordinates *x* and *y*, the system covariance matrix is shown in (5) and the observation error covariance matrix in (6).

(5) $$\begin{array}{c} Q=q^{T}q\\ q=\left[\begin{array}{ccc} \begin{array}{cc} \frac{T^{3}\sigma_{Jx}}{3} & \frac{T^{2}\sigma_{Jx}}{2} \end{array} & \begin{array}{cc} T\sigma_{Jx} & \frac{T^{3}\sigma_{Jy}}{3} \end{array} & \begin{array}{cc} \frac{T^{2}\sigma_{Jy}}{2} & T\sigma_{Jy} \end{array} \end{array}\right] \end{array}$$

(6) $$R=\left[\begin{array}{cc} \sigma_{x}^{2} & 0\\ 0 & \sigma_{y}^{2} \end{array}\right]$$

#### 4.8.3. Statistical Parameters

The parameters σ*_Jx_* and σ*_Jy_* belongs to the random nature of the system, for this reason, they were extracted from experimental data. A number *c* = 15 of cells were randomly selected and manually tracked, through *p* = 100 frames, to register their vector positions, *x* and *y*. Three numerical derivatives were performed in order to get the jerk vector for each dimension, then the standard deviation of each jerk vector was computed. Finally, σ*_Jx_* is the average of those σ*_Jx__c_*, see (7) and (8). The same process was done for σ*_Jy_*.

(7) $$\sigma_{Jx_{c}}^{2}=\frac{1}{p-1}\overset{p}{\underset{i=1}{\sum }}{\left(Jx_{i}-{\bar{J}}_{x}\right)}^{2}$$

(8) $$\sigma_{Jx}=\frac{1}{c}\overset{c}{\underset{n=1}{\sum }}\sigma_{Jx_{n}}$$

The state estimation with prediction and correction is performed in every iteration once the current cell has been found with the aim of updating the second and third order derivatives because a single prediction is used only when intersection scheme fails to find the selected cell. For the observation error covariance matrix, the standard deviations were set in such a way that the estimates were very close to measurements and that the influence of Q was incorporated to avoid singularities in the calculus. The parameters are shown in Table 5.

A set of artificial images was generated in order to test the Kalman filter estimation. The image sequence was designed to simulate the trajectory of a cell, the coordinates of the cell were determined mathematically as *x* = *c* and *y* = *A*cos(*2π f t*), where *c* is a constant or a bias, *f* = 4, *A* was adjusted to produce a 36 pixels peak to peak amplitude and *t* is a vector with the same number of elements than *x*. The area of the object in the images was the average cells area obtained from the experimental data.

**Figure 8:**
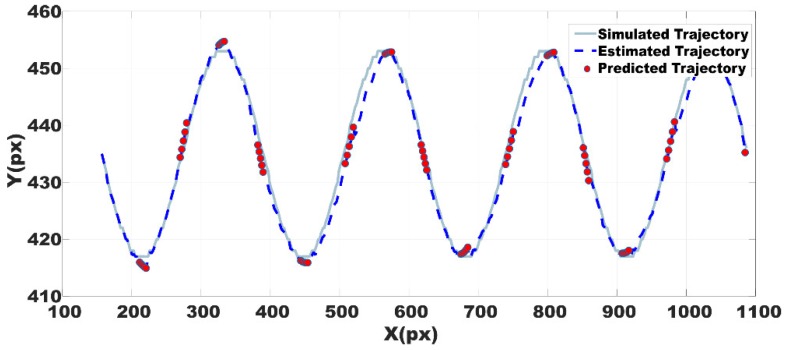
Kalman filter testing. Simulated, estimated and predicted trajectories in solid, dashed and dotted lines, respectively. Position in X and Y axes respectively, measured in pixels (px).

The estimation was obtained with Kalman filter configured with the statistical parameters described above. In Figure 8 it can be seen the trajectory estimation using the whole scheme predictor--corrector. As some cells disappear during some frames, statistical measurements for Kalman correction are not available along the entire image sequence. Thus, several missed points were used as a focused test of the Kalman prediction, that is, with no evaluation of correction equations.

The mean square error between trajectories in both cases is reported in Table 6. By using the centroid as the position of the cell, the reported error is tolerable since it falls inside the average ROI area and it is possible to find again the selected cell. Thus, the Kalman filter has a good performance.

**Figure 9:**
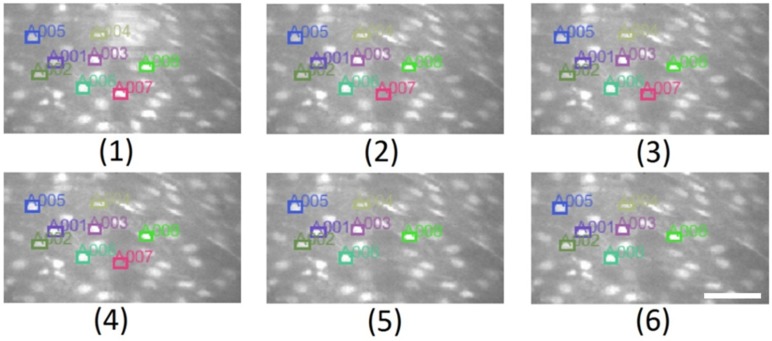
Tracking results through six consecutive frames. Eight cells were selected by the user, only ONLINE cells are labeled, ROI surrounded and tracked. OFFLINE cells are not tracked as shown with cell A007; the cell is visible at frame (**1**), then it vanishes as of frame (**2**) and its reliability counter increases by one between frames (**2**–**4**). At frame (**4**) its reliability counter is equal to RL = 3, then it is tagged as OFFLINE and is not tracked as of frame (**5**). Frame (**6**) shows the tracking of remaining ONLINE cells. Bar length is 40 µm.

Tracking results with RL = 3 are shown in Figure 9. It can be seen that the value of reliability counter of cell “A007” increased to three, from frame 1 to 4, then its status changed to OFF-LINE and the cell “A007” is not tracked anymore as of frame 5.

### 4.9. Feature Extraction and Data Storage

This is the last block of the proposed solution. The block receives as input the *new tracking label*. The cell under investigation is identified with that label, the ROI is relocated and the intensity average is computed from the respective ratio frame, that is, the CP is extracted. In this stage, more features can be extracted from the binarized image. Online Supplementary Video 3 shows the analysis complete flow on the software application developed with the approach described throughout this manuscript. This system has, therefore, the potential to help health experts to expand their studies by greatly limiting the movement artefacts due to the manipulation of the extracellular solution or due the delivery of a mechanical stimulus.

## Figures and Tables

**Table 1:** Mean of CPs obtained using the proposed approach ($\bar{\mathrm{CP}}$) and the mean square error (MSE) between CPs extracted using ImageJ-Supervised Tracking and our automatic tracking proposal. Adenosine triphosphate (ATP) stimulus experiment.

Cell	$\bar{\mathrm{CP}}$	MSE	Cell	$\bar{\mathrm{CP}}$	MSE
1	0.9098	1.6242 × 10^−4^	9	0.8851	2.9395 × 10^−5^
2	1.0111	4.4606 × 10^−4^	10	0.8607	5.5402 × 10^−5^
3	0.9515	6.0467 × 10^−5^	11	0.8728	7.9154 × 10^−5^
4	0.9490	4.7925 × 10^−5^	12	0.8504	1.1613 × 10^−4^
5	0.8713	1.2525 × 10^−5^	13	0.8498	1.3611 × 10^−5^
6	0.9013	1.7247 × 10^−5^	14	0.8648	8.0574 × 10^−5^
7	0.8699	2.6627 × 10^−5^	15	0.8851	2.2170 × 10^−5^
8	0.8481	3.4207 × 10^−5^	-	-	-

**Table 2:** Average performance results with three different reliability limit (RL) values.

RL	$\rho_{\max}$ (%)	$\rho_{\min}$ (%)	$\rho_{\mathrm{mean}}$ (%)
3	104.3956	67.1378	84.8415
5	104.3956	76.4045	89.4309
7	104.3956	79.7753	92.3110

**Table 3:** Mean of CPs obtained using the proposed approach ($\bar{\mathrm{CP}}$) and the mean square error (MSE) between CPs extracted using ImageJ-Supervised Tracking and our automatic tracking proposal. Mechanical stimulus experiment.

Zone	Cell	$\bar{\mathrm{CP}}$	MSE
1	1	0.8991	3.5821 × 10^−6^
	2	0.8765	4.5569 × 10^−6^
	3	0.8937	3.2133 × 10^−5^
2	4	1.0551	1.6675 × 10^−3^
	5	1.0483	4.2171 × 10^−4^
	6	1.0488	9.2146 × 10^−4^
3	7	0.9941	3.9861 × 10^−6^
	8	0.9676	2.2033 × 10^−5^
	9	1.0074	8.7559 × 10^−5^

**Table 4:** Average performance results with different RLs.

RL	$\rho_{\max}$ (%)	$\rho_{\min}$ (%)	$\rho_{\mathrm{mean}}$ (%)
3	100.7117	58.0756	69.9652
5	101.0714	59.7938	73.1079
7	101.0714	61.1684	75.1659

**Table 5:** Statistical parameters for Kalman filter.

Parameter	σ*_Jx_*	σ*_Jy_*	σ*_x_*	σ*_y_*
Value	0.0269	0.0993	0.001	0.001

**Table 6:** Mean square error between trajectories.

Estimator Configuration	MSE*_x_*	MSE*_y_*
Prediction-Correction	9.6537	0.8958
Only Prediction	10.5347	0.8929

## Supplementary Materials

The supplementary materials are available online at http://www.mdpi.com/1422-0067/19/11/3440/s1.

## Abbreviations

ATP	Adenosine trisphosphate
CP	Calcium profile
Ca^2+^	Divalent calcium ion
[Ca^2+^]_i_	Intracellular Ca^2+^ concentration
ECs	Endothelial cells
EDH	Endothelial-dependent hyperpolarization
NO	Nitric oxide
eNOS	Nitric oxide synthase
PSS	Physiological salt solution
ROI	Region of interest
RL	Reliability limit
VSMC	Vascular smooth muscle cell
MSE	Mean square error
