# Supplementary figures and images for: Automated Intracellular Calcium Profiles Extraction from Endothelial Cells Using Digital Fluorescence Images
Source: Int J Mol Sci. 2018 Nov 2;19(11):3440. doi: 10.3390/ijms19113440 (PMC6274978; doi:10.3390/ijms19113440)

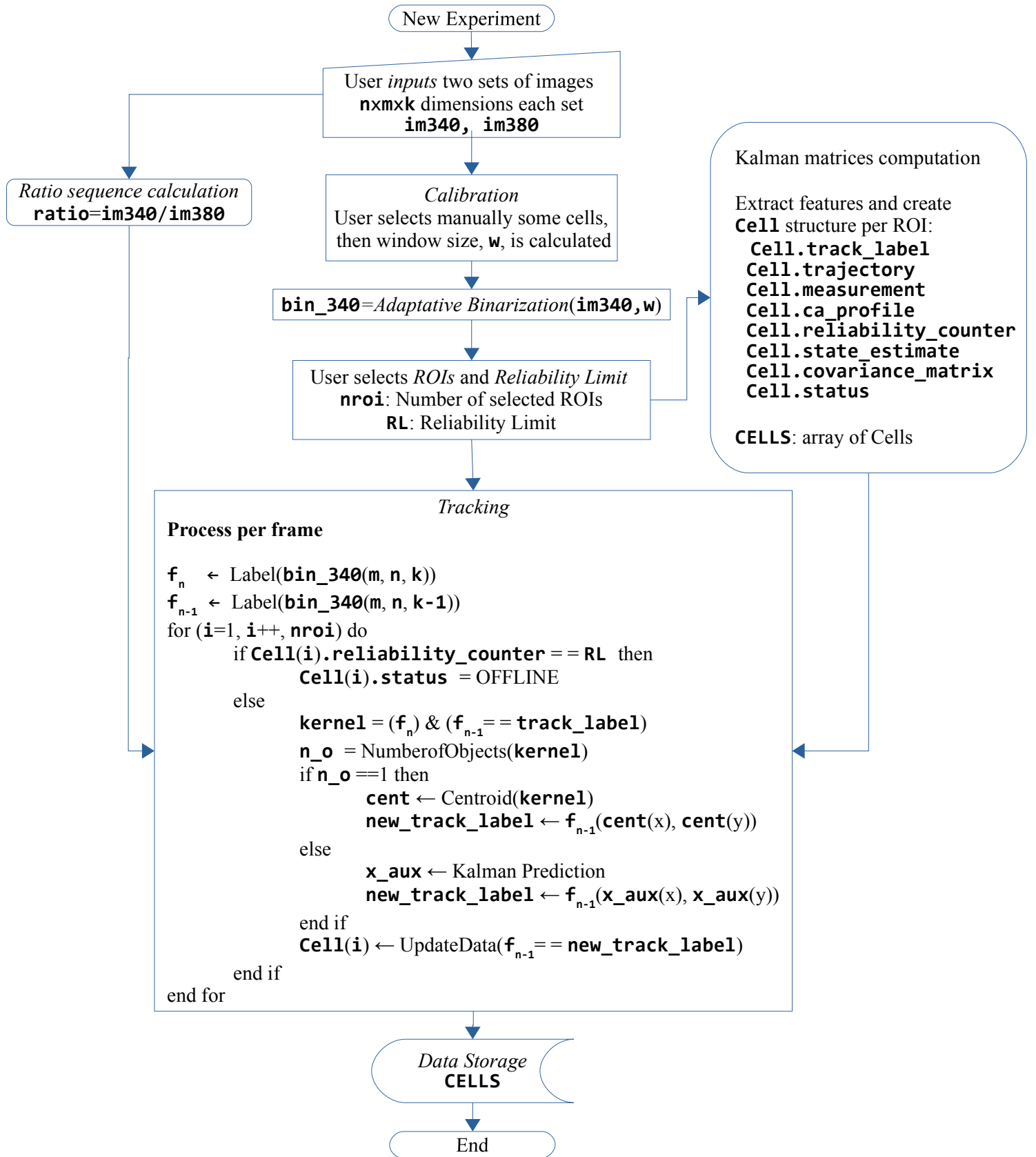

Supplement: Supplementary file 1 [file ijms-19-03440-s001.zip › Flowchart.pdf]
